# Unterschiedliche anatomische Spezifitäten des residualen PASI bei Biologikatherapien der Psoriasis

**DOI:** 10.1111/ddg.15924_g

**Published:** 2026-05-05

**Authors:** Martina Burlando, Irene Schiavetti, Aurora Parodi, Emanuele Cozzani, Luca Mastorino, Eleonora Bongiovanni, Ilaria Salvi, Martina Del Vecchio, Paolo Dapavo

**Affiliations:** ^1^ Clinica Dermatologica DissaL Ospedale Policlinico San Martino – IRCCS Genova Italy; ^2^ Section of Biostatistics Department of Health Sciences University of Genova Genova Italy; ^3^ Section of Dermatology Department of Medical Sciences University of Turin Turin Italy

**Keywords:** biologische Therapie, Psoriasis, residualer PASI, Biologics, Psoriasis, residual PASI

## Abstract

**Hintergrund und Ziele:**

Ein besseres Verständnis der Immunpathogenese der Psoriasis hat zur Entwicklung effektiver zielgerichteter Therapien geführt. Allerdings weisen Patienten, die ohne vollständige Abheilung auf die Behandlung ansprechen, eine Resterkrankung auf. Das Ziel dieser Studie war es, spezifische anatomische Lokalisationen der residualen Erkrankung (oder des residualen *Psoriasis Area Severity Index* [PASI]) in Bezug auf verschiedene Biologika nachzuweisen.

**Methoden und Patienten:**

In dieser retrospektiven, multizentrischen Beobachtungsstudie wurden die klinischen Daten von Patienten mit Psoriasis analysiert, die mindestens 6 Monate lang mit Biologika behandelt worden waren. Die Datenanalyse konzentrierte sich auf Patienten mit residualem PASI.

**Ergebnisse:**

Insgesamt 228 von 1.000 Patienten wiesen trotz Erreichen eines PASI 90 in den Wochen 24 bis 28 der Biologikatherapie eine Resterkrankung auf. Die am häufigsten von der residualen Erkrankung betroffenen anatomischen Lokalisationen waren die unteren Extremitäten (44,3%). Wir beobachteten Unterschiede zwischen den Biologika hinsichtlich der Häufigkeit und Lokalisation der Erkrankung. Die Lokalisation residualer Hautläsionen an den Beinen stand im Zusammenhang mit einem Wechsel oder einer Unterbrechung der Behandlung. Die Wirkstoffe mit dem höchsten beziehungsweise niedrigsten Anteil an Patienten mit residualer Erkrankung an den unteren Extremitäten waren Secukinumab und Risankizumab.

**Schlussfolgerungen:**

Die Behandlung mit Anti‐IL‐17‐ und Anti‐IL‐23‐Medikamenten ist durch die Persistenz residualer Läsionen gekennzeichnet, wobei Unterschiede hinsichtlich der Häufigkeit und der anatomischen Lokalisationen bestehen.

## EINLEITUNG

Psoriasis ist eine chronisch‐inflammatorische Hauterkrankung, von der 2% bis 3% der Weltbevölkerung betroffen sind.^1^, ^2^ Sie ist durch unkontrollierte Aktivierung sowohl der adaptiven als auch der angeborenen Immunität gekennzeichnet, was zur Überproduktion proinflammatorischer Zytokine wie Tumornekrosefaktor (TNF)‐α und Interleukin (IL)‐17 führt.^3^ Aufgrund der Behandlungsfortschritte können viele Patienten eine nahezu vollständige Abheilung (*total skin clearance*; TSC) erreichen,^4^ doch diejenigen, bei denen keine TSC eintritt, leiden häufig unter negativen Auswirkungen auf ihre gesundheitsbezogene Lebensqualität (HRQoL) sowie einem erhöhten Risiko für Begleiterkrankungen wie Psoriasis‐Arthritis.^5^ Das Verständnis der spezifischen Lokalisationen der mit verschiedenen biologischen Wirkstoffen assoziierten residualen Erkrankungen kann dabei helfen, Therapieentscheidungen effektiv zu treffen.

## MATERIALIEN UND METHODEN

Diese Beobachtungsstudie analysierte Daten von Patienten mit Psoriasis, die im Jahr 2022 an die Dermatologischen Abteilungen des Ospedale Policlinico San Martino und der Universität Turin, Italien, überwiesen wurden. Insgesamt 1000 Patienten erhielten mindestens 6 Monate lang entweder Anti‐IL‐17‐Behandlungen (Secukinumab, Ixekizumab, Brodalumab, Bimekizumab) oder Anti‐IL‐23‐Behandlungen (Guselkumab, Risankizumab, Tildrakizumab). Der Schwerpunkt lag auf Patienten mit einem posttherapeutischen residualen *Psoriasis Area Severity Index* (PASI), wobei die Lokalisation der Hautläsionen und unterschiedliche Ergebnisparameter, darunter der Zeitpunkt des Erreichens eines PASI 90, untersucht wurden.

## ERGEBNISSE

Von den 1000 Patienten wiesen 228 nach der Behandlung noch Restläsionen auf, während 772 einen PASI 100 erreichten und daher aus der Studie ausgeschlossen wurden. Unter den Patienten mit residualen Läsionen hatten 61,8% Begleiterkrankungen (Komorbidität) und das Durchschnittsalter bei der Diagnose betrug 38,6 Jahre. Die demografischen Daten, Gewohnheiten und die Komorbidität sind in Tabelle 1 zusammengefasst.

**TABELLE 1:** Demografische Daten, Gewohnheiten und Komorbidität (n = 228).

**Ethnizität**	Weiß	218 (95,6%)
	Schwarz	3 (1,3%)
	Andere	7 (3,1%)
**Geschlecht**	Weiblich	82 (36,0%)
	Männlich	146 (64,0%)
**Alter, Jahre**		53,3 ± 15,34 (19,0 – 89,0)
**BMI**		26,4 ± 4,94
**Alkohol**	Nie getrunken	62 (27,2%)
	Gelegentlicher Konsum	99 (43,4%)
	Regelmäßiger Konsum	26 (11,4%)
	Nicht angegeben	41 (18,0%)
**Rauchen**	Nie geraucht	71 (31,1%)
	Ehemaliger Raucher	58 (25,4%)
	Raucher	99 (43,4%)
**Komorbidität**		
	Mindestens eine Begleitkrankheit	141 (61,8%)
	Autoimmunerkrankung	Unbehandelt 1 (0,4%) Behandelt 9 (3,9%)
	Zerebrovaskuläre Erkrankung	Unbehandelt 0 (0,0%) Behandelt 5 (2,2%)
	Kardiovaskuläre Erkrankung	Unbehandelt 1 (0,4%) Behandelt 45 (19,7%)
	Chronische Nierenerkrankung	Unbehandelt 0 (0,0%) Behandelt 2 (0,9%)
	Gastrointestinale Erkrankung	Unbehandelt 5 (2,2%) Behandelt 11 (4,8%)
	HIV	Unbehandelt 0 (0,0%) Behandelt 0 (0,0%)
	HBV	Unbehandelt 1 (0,4%) Behandelt 5 (2,2%)
	Hämatologische Erkrankung	Unbehandelt 3 (1,3%) Behandelt 5 (2,2%)
	Hypertension	Unbehandelt 2 (0,9%) Behandelt 62 (27,2%)
	Maligner Tumor	Unbehandelt 0 (0,0%) Behandelt 12 (5,3%)
	Metabolische Erkrankung	Unbehandelt 6 (2,6%) Behandelt 53 (23,2%)
	Muskuloskelettale Erkrankung	Unbehandelt 2 (0,9%) Behandelt 11 (4,8%)
	Neurologische Erkrankung	Unbehandelt 0 (0,0%) Behandelt 2 (0,9%)
	Psychiatrische Erkrankung	Unbehandelt 0 (0,0%) Behandelt 8 (3,5%)
	Andere	Unbehandelt 5 (2,2%) Behandelt 31 (13,6%)

**ABBILDUNG 1:**
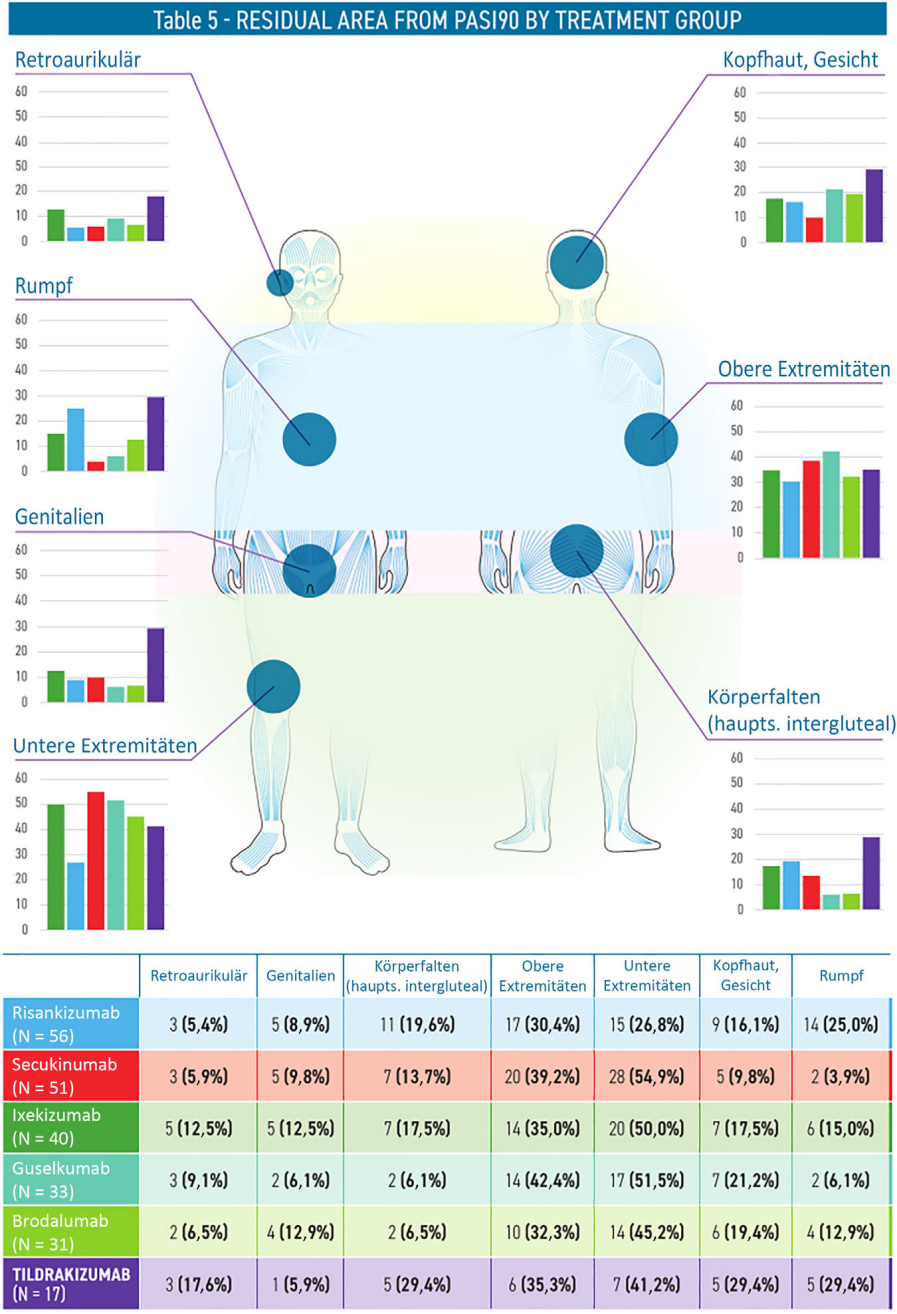
Bereiche residualer Erkrankung nach PASI 90 nach Therapiegruppe.

Ein PASI‐90‐Ansprechen wurde im Mittel nach 19,5 Wochen erreicht. Zu den am stärksten betroffenen Bereichen gehörten die unteren Extremitäten (44,3%), die oberen Extremitäten (35,5%) und die Kopfhaut/das Gesicht (17,1%). Bemerkenswert ist, dass Secukinumab die höchste Inzidenz residualer Läsionen an den Beinen (54,9%) aufwies, während Risankizumab und Tildrakizumab mit einer höheren Prävalenz von Läsionen am Rumpf assoziiert waren (Abbildung 1).

Siebzehn Prozent der Patienten wechselten ihre Behandlung, primär auf ärztliche Empfehlung (84,6%). Läsionen an den unteren Extremitäten waren mit einer höheren Rate an Therapieänderungen verbunden (22,8%). Brodalumab und Risankizumab waren die am häufigsten gewählten Optionen für einen Therapiewechsel. Zu den Faktoren, die diese Behandlungswechsel beeinflussten, gehörten frühere Behandlungen mit Secukinumab und Ixekizumab. Interessanterweise bewerteten die Patienten ihre Akzeptanz von Restläsionen mit 83,1 ± 17,88, wobei 85,0% angaben, dass die residuale Erkrankung keine Medikationsänderung erforderlich machte. Die klinischen Merkmale und die Behandlung von Patienten, die einen PASI 90 erreichten, sind in Tabelle 2 zusammengefasst.

**TABELLE 2:** Klinische Merkmale und Behandlung von Patienten, die einen PASI 90 erreichten.

PASI zu Beginn der letzten Behandlung		14,8 ± 5,90 (5,0–37,0)
PGA zu Beginn der letzten Behandlung	Abgeheilt	0 (0,0%)
	Nahezu abgeheilt	1 (0,4%)
	Mild	4 (1,8%)
	Mäßig	85 (37,3%)
	Mäßig bis schwer	95 (41,7%)
	Schwer	43 (18,9%)
Konkomitante topische Behandlung	Kein Topikum	150 (65,8%)
	Clobetasol‐Schaum	7 (3,1%)
	Clobetasol + Calcipotriol‐Schaum	56 (24,6%)
	Clobetasol + Calcipotriol‐Gel	15 (6,6%)
Behandlungswoche, in der PASI 90 erreicht wurde		19,5 ± 9,53 (4,0 ‐ 28,0)
PGA bei Erreichen von PASI 90	Abgeheilt	161 (70,6%)
	Nahezu abgeheilt	64 (28,1%)
	Schwach	2 (0,9%)
	Mäßig	0 (0,0%)
	Mäßig bis schwer	1 (0,4%)
	Schwer	0 (0,0%)
Residualer Bereich	Retroaurikulär	19 (8,3%)
	Genitalien	22 (9,6%)
	Falten (haupts. intergluteal)	34 (14,9%)
	Obere Extremitäten	81 (35,5%)
	Untere Extremitäten	101 (44,3%)
	Kopfhaut, Gesicht	39 (17,1%)
	Rumpf	33 (14,5%)
Therapiewechsel oder ‐unterbrechung		39 (17,1%)
Für wen die erste Wahl?	Arzt	33 (84,6%)
	Patient	6 (15,4%)
Neue Therapie	Brodalumab	10 (25,6%)
	Risankizumab	9 (23,1%)
	Ixekizumab	8 (20,5%)
	Keine Therapie	5 (12,8%)
	Bimekizumab	2 (5,1%)
	Certolizumab pegol	1 (2,6%)
	Secukinumab	1 (2,6%)
	Guselkumab	1 (2,6%)
	Tildrakizumab	1 (2,6%)
	Ustekinumab	1 (2,6%)
Inwieweit akzeptierte der Patient seinen residualen PASI‐Zustand?		83,1 ± 17,88 (5,0–100,0)
Motivierte der residuale PASI an dieser Lokalisation den Patienten, das Medikament zu wechseln?	Nein	192 (85,0%)
	Ja	34 (15,0%)

## DISKUSSION

Unsere Studie ist die erste, die den Zusammenhang zwischen einzelnen biologischen Wirkstoffen der Klassen Anti‐IL‐17 und Anti‐IL‐23 und den Lokalisationen der residualen Erkrankung (Rest‐PASI) nach Erreichen von PASI 90 untersuchte. Wir bemerkten, dass Secukinumab die höchste Prävalenz (über 54%) von Resterkrankungen in den unteren Extremitäten aufwies, was mit dem Wechsel oder der Unterbrechung der Therapie signifikant assoziiert war. Ixekizumab und Brodalumab folgten mit 50,0% und 45,2%, während Risankizumab und Tildrakizumab die niedrigsten Prävalenzen aufwiesen (26,8% und 41,2%).

Die geringeren Raten an residualen Erkrankungen unter Risankizumab und Tildrakizumab könnten mit der Rolle von IL‐23 bei der Differenzierung von T‐Helferzellen zusammenhängen.^6^ Frühere Daten haben darauf hingedeutet, dass Restläsionen in besser sichtbaren Bereichen die von den Patienten berichteten Ergebnisse negativ beeinflussen.^7^

Bemerkenswert war hingegen, dass in unserer Untersuchung die auf die Beine lokalisierte PASI‐Resterkrankung der einzige anatomische Faktor war, der mit einer höheren Wahrscheinlichkeit einer Behandlungsänderung assoziiert war. Dies könnte darauf hindeuten, dass Patienten mit Läsionen an den unteren Extremitäten auch bei einem PASI 90 eine erhebliche Krankheitslast aufweisen.

Mashiko et al. stellten fest, dass Restplaques, obwohl sie gut auf die Behandlung ansprechen, immer noch molekulare Unterschiede zu unbehandelten Plaques aufweisen, Dies weist auf wichtige Psoriasis‐Signalwege und eine Resistenz gegen die Remission hin.^8^

In unserer Studie gaben 85% der Patienten an, dass ein residualer PASI sie nicht zu einem Medikamentenwechsel veranlassen würde, und die meisten akzeptierten die Persistenz ihrer Erkrankung. Dies steht im Gegensatz zu anderen Untersuchungen, die feststellten, dass selbst minimale Hautläsionen die Lebensqualität beeinträchtigen können, was sich in *Dermatology Life Quality Index* (DLQI)‐Werten von mehr als 1 widerspiegelt, wobei 20% der Patienten, die eine TSC erreichten, dennoch über negative Auswirkungen der Psoriasis berichteten.^7^

Ein Drittel der Patienten in unserer Studie war biologikanaiv, was zu verbesserter Wirksamkeit der Behandlung führen kann. Randomisierte kontrollierte Studien zu IL‐17/IL‐23‐Inhibitoren haben bessere patientenberichtete Ergebnisse bei biologikanaiven Personen gezeigt.^7^

Die meisten Patienten in unserer Studie akzeptierten residuale Hautläsionen, wobei nahezu 85% der Therapieänderungen interessanterweise von Ärzten vorgenommen wurden. Dies zeigt möglicherweise eine Diskrepanz zwischen der ärztlichen und Patientenwahrnehmung der Krankheitslast auf.

Die Patienten erreichten nach einer medianen Behandlungsdauer von 19,5 Wochen eine Verbesserung des PASI‐Scores um ≥ 90%, wobei es Variabilität zwischen den biologischen Wirkstoffen gab. Die Wirkgeschwindigkeit ist für Patienten mit Akutschüben von entscheidender Bedeutung, während solche mit einer stabilen Krankheitsgeschichte möglicherweise langsamere Behandlungen zugunsten einer besseren Sicherheit akzeptieren.

Unsere Studie konzentrierte sich auf Patienten mit residualem PASI, was zu einem Selektionsbias und einer eingeschränkten Repräsentativität geführt haben könnte, da sie nur an zwei Universitätszentren in Italien durchgeführt wurde. Obwohl die retrospektive Datenerhebung eine Einschränkung darstellte, können die Erkenntnisse bei der Integration von Ergebnissen im Zusammenhang mit residualen Hautläsionen in die klinische Leitlinienpraxis wertvoll sein.

## DANKSAGUNG

Open access publishing facilitated by Universita degli Studi di Genova, as part of the Wiley ‐ CRUI‐CARE agreement.

## INTERESSENKONFLIKT

M.B. war als Sprecherin für AbbVie, Almirall, Amgen, Eli Lilly, Janssen, Novartis und UCB tätig. A.P. war als Sprecherin für AbbVie, Almirall, Eli Lilly, Janssen, Novartis und UCB tätig. E.C. war als Sprecher für AbbVie, Almirall, Eli Lilly und Novartis tätig. L.M. war als Sprecher für AbbVie, Leo Pharma, Accord und Almirall tätig. P.D. war als Sprecher für AbbVie, Almirall, Amgen, Eli Lilly, Janssen, Novartis und UCB tätig.
